# Beneficial Effects of Silybin Treatment After Viral Eradication in Patients With HCV-Related Advanced Chronic Liver Disease: A Pilot Study

**DOI:** 10.3389/fphar.2022.824879

**Published:** 2022-02-02

**Authors:** Valentina Cossiga, Marco Sanduzzi-Zamparelli, Victor Sapena, Maria Guarino, Marcello Dallio, Emanuele Torrisi, Luca Pignata, Alessandro Federico, Federico Salomone, Filomena Morisco

**Affiliations:** ^1^ Department of Clinical Medicine and Surgery, Gastroenterology and Hepatology Unit, University of Naples “Federico II”, Naples, Italy; ^2^ Medical Statistics Core Facility, Institut D'Investigacions Biomédiques August Pi i Sunyer (IDIBAPS), Hospital Clinic Barcelona, Barcelona, Spain; ^3^ Department of Precision Medicine, Hepato-Gastroenterology Unit, University of Campania “Luigi Vanvitelli”, Naples, Italy; ^4^ Division of Gastroenterology, Acireale Hospital, Azienda Sanitaria Provinciale di Catania, Catania, Italy

**Keywords:** HCV, DAA, silybin, transient elastography, advanced liver disease

## Abstract

**Introduction and Aims:** HCV eradication by direct-acting antivirals (DAAs) improves liver outcomes and reduces overall liver mortality. However, patients with advanced chronic liver disease (ACLD) may experience a progression of liver disease despite viral clearance. Silybin has shown hepatoprotective effects in experimental models, but clinical data are limited. The aim of this study is to evaluate the effect of a highly bioavailable form of silybin on liver fibrosis in patients with HCV-related ACLD after viral eradication with DAAs, in comparison with the standard of care.

**Methods:** In this multicenter and prospective study, HCV patients with ACLD achieving SVR12 were treated with the combination of silybinphospholipid complex with vitamin D and vitamin E (Realsil 100D^®^, Ibi Lorenzini S.p.A., Aprilia, Italy) for 12 months (R group) compared to controls (C group). Patients were submitted to transient elastography (TE) and to the enhanced liver fibrosis (ELF) test at baseline, week 24, and week 48.

**Results:** One hundred sixteen patients were enrolled, 56 in the R group and 60 in the C group. The median age was 68 years, and 53% were male, with no differences between groups. In both groups, liver stiffness improved at 6 and 12 months compared to baseline. However, patients in the R group compared to those in the C group showed a higher reduction of liver stiffness after 6 months (−2.05, 95% CI −3.89 to −0.22, *p* < 0.05) and 12 months of treatment (−2.79, 95% CI −4.5 to −1.09, *p* < 0.01) in comparison with baseline. No significant difference in the reduction of ELF was observed between the two groups. During the follow-up, four patients developed HCC, all in the C group.

**Conclusions:** In HCV-related ACLD, the hepatoprotective effects of silybin may represent a tool to counteract liver disease progression.

## Introduction

More than 180 million people worldwide are infected with hepatitis C virus (HCV), which still remains a major cause of chronic liver disease (Thrift et al., 2017). Over the last few years, direct-acting antivirals (DAAs) became available for HCV treatment, with high rates (>95%) of sustained virological response (SVR), regardless of the stage of fibrosis and viral genotype (Ponziani et al., 2017). Notably, SVR achievement is associated with improvement of liver function, regression of fibrosis, reduction of portal hypertension, and an overall decrease of liver-related events and mortality (Innes et al., 2017; Carrat et al., 2019; Morisco et al., 2021). However, patients with advanced chronic liver disease (ACLD) may experience a progression of liver disease despite viral clearance (Carrat et al., 2019). For this reason, the assessment of liver fibrosis prior to and after antiviral treatment is fundamental to determining the prognosis and establishing an adequate follow-up (Singal et al., 2010). The most widely used and validated technique, in this setting of patients, is transient elastography (TE) which is the non-invasive method with highest accuracy to identify the advanced stage of fibrosis (European Association for the Study of the Liver 2021).

Silymarin is the seed extract of milk thistle (*Silybum marianum*) consisting of seven flavonolignans and taxifolin, which is widely used in a variety of chronic liver diseases for its anti-inflammatory, anti-fibrotic, and antioxidant effects (Federico et al., 2017). Studies in experimental models of liver disease have shown that silybin, the main component of silymarin, may be considered an anti-fibrotic molecule inhibiting transforming growth factor β (TGFβ) and platelet-derived growth factor (PDGF) cascades (Muriel et al., 2005). Besides, it inhibits the pro-inflammatory signals involved in the synthesis of cytokines such as tumor necrosis factor α (TNF-α) and in the modulation of apoptosis modulating levels of bcl-2–like protein-4 (Bax), while the antioxidant effect is mainly related to its ability to act as a free radical scavenging and lipid peroxidation inhibitor (Loguercio and Festi, 2011; Salomone et al., 2016; Abenavoli et al., 2018). Furthermore, clinical studies suggest that the oral administration of silybin in patients with liver diseases is able to reduce hepatic inflammation translating in a reduction of serum transaminases with an excellent safety profile (Lozano-Sepulveda et al., 2015).

In this scenario, the aim of the present study is to evaluate the effects of a combination of silybinphospholipid complex with vitamin D and vitamin E (Realsil 100D^®^, Ibi Lorenzini S.p.A., Aprilia, Italy) on the modulation of liver fibrosis in comparison with the control group of patients with HCV-related ACLD achieving SVR after DAA treatment.

## Materials and Methods

### Patients and Study Design

This is a multicenter, prospective, controlled, and interventional study assessing the effects of an oral combination of silybinphospholipid complex with vitamin D and vitamin E (Realsil 100D^®^, Ibi Lorenzini S.p.A., Aprilia, Italy) in patients with HCV-related ACLD who achieved SVR after DAAs. The study was conducted in three centers in Southern Italy (Department of Clinical Medicine and Surgery of Federico II University of Naples, Division of Gastroenterology of Acireale Hospital, and Hepato-Gastroenterology Unit of University of Campania “Luigi Vanvitelli”). The ethics committee of Federico II University approved the study protocol (no. 34/2016), and all patients signed an informed written consent form.

Inclusion criteria for the study were 1) severe fibrosis, before starting antiviral treatment, defined by liver stiffness measurement (LSM) ≥9.5 kPa (corresponding to ≥F3 according to the METAVIR score) and/or radiological/clinical signs of cirrhosis and 2) confirmed SVR at 12 weeks after the end of antiviral treatment. Exclusion criteria were 1) Child–Pugh score >6 points; 2) active or previous history of hepatocellular carcinoma (HCC); 3) prior liver transplantation; 4) concomitant coinfection with hepatitis B virus (HBV) and/or human immunodeficiency virus (HIV); 5) other causes of liver disease; 6) decompensated diabetes mellitus; or 7) body mass index (BMI) > 30.

**FIGURE 1:**
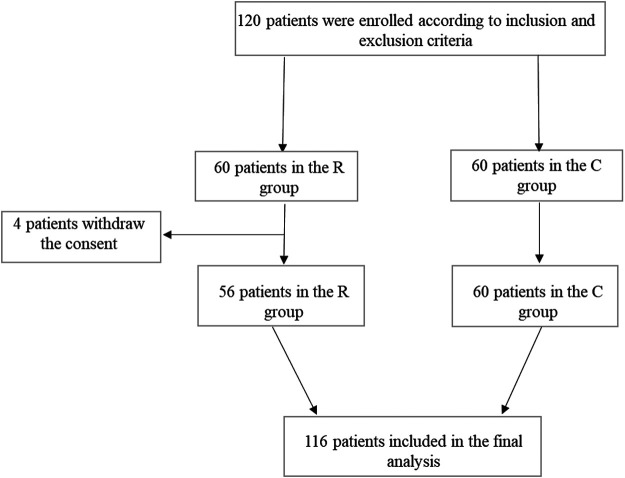
Study flowchart.

Between January 2016 and December 2017, 120 patients were enrolled according to inclusion and exclusion criteria. Sixty patients were randomized in the R group and 60 in the C group. Four patients in the R group withdrew the informed consent before starting the treatment, and 116 patients were considered for the final analysis (56 in the R group and 60 in the C group). A study flowchart is depicted in Figure 1.

At baseline (SVR12), patients in the R group received active treatment with Realsil 100D^®^ (303 mg of silybinphospholipid complex, 10 μg of vitamin D, and 15 mg of vitamin E) twice a day for 12 months. In the control group (C), patients underwent no intervention and continued standard follow-up after SVR.

Demographic and clinical data (age, gender, body mass index (BMI), diabetes mellitus, arterial hypertension, history of previous cancer), HCV genotype, type of DAA therapy, and laboratory parameters (platelet count, transaminases) were collected at study inclusion. SVR was defined as undetectable HCV RNA at 12 weeks after the end of treatment. Laboratory and clinical parameters were repeated at 6 and 12 months after the enrollment. Liver-related events were collected until death, until withdrawal of informed consent, or until November 20, 2020.

### Non-Invasive Assessment of Liver Fibrosis

Liver fibrosis was evaluated non-invasively with both TE and enhanced liver fibrosis (ELF) test at baseline (SVR12) and at 6 and 12 months. Patients were submitted to TE and to a fasting blood sample in the same day. Liver stiffness measurements (LSMs) were performed by a single well-trained operator in each center using FibroScan^®^ 502 Touch (EchoSens, Paris, France). The results were expressed in kilopascal (kPa) in the range from 2.5 to 75 kPa. The IQR was defined as an index of intrinsic variability of LSM. Only those measurements with more than ten successful acquisitions, with a success rate of at least 60% and an interquartile range lower than 30%, were classified as valid and taken into consideration for statistical evaluation (Nitta et al., 2009).

For the ELF test, fasting blood samples were obtained in the same day of LSM. All sera were frozen and stored at −20°C until determination. Samples were assayed in an automated analyzer that performs magnetic separation enzyme immunoassay tests (ADVIA Centaur; Siemens Healthcare Diagnostics, Tarrytown, NY). The results were entered into the manufacturer’s published algorithm to derive an ELF score [ELF = 2.278 + 0.851 ln(HA)+ 0.751 ln(PIIINP)+0.394 ln(TIMP-1)].

### Statistical Analysis

Quantitative variables were expressed as median and interquartile range [IQR 25th–75th percentiles]. Categorical variables were described as absolute frequencies and percentages (%).Fisher’s exact test was used to compare categorical variables, and for quantitative or ordinal variables, we used non-parametric methods: Mann–Whitney (two groups) for independent data and Wilcoxon signed-rank test (two groups) for dependent data.

Liver stiffness variations between treatment and placebo groups were estimated, compared between baseline (SVR12) and the end of treatment, by multivariable linear mixed models for repeated measurements (MMRMs), using age, gender, cirrhosis, ALT, and AST as adjusting factors. The reported *p*-values for MMRMs were calculated with a non-parametric approach, obtained from the same model but using ranges of liver stiffness instead of raw values.

All statistical analyses were performed using SAS software v9.4^®^, and we considered a two-sided type I error as 5%.

## Results

Table 1 summarizes the baseline characteristics of the enrolled patients. Overall, 53% were male, with a median age of 68 [IQR 60–72] years and a median BMI of 25.8 [IQR 23.6–29.1] kg/m^2^. There were no significant differences between groups regarding age, sex, and BMI.

**TABLE 1:** Baseline characteristics of patients at study inclusion.

	Overall (*n* = 116)	R group (*n* = 56)	C group (*n* = 60)	*p*-Value
Age (years), median [IQR]	68 [60–72]	68 [59.5–71.5]	67.5 [60–73.5]	0.8595
Gender (male), n (%)	62 (53.4)	30 (53.6)	32 (53.3)	0.9999
BMI (kg/m^2^), median [IQR]	25.8 [23.6–29.1]	25.5 [23.7–28.1]	25.9 [23.1–27.6]	0.8531
ELF, median [IQR]^a^	10.7 [9.6–11.2]	10.7 [10–11.2]	10.7 [9.5–11.2]	0.6120
TE (kPa), median [IQR]	9.6 [7.2–14]	10.2 [7.2–15]	9.1 [7.3–12]	0.4657
History of AHT (yes), n (%)	34 (29.3)	18 (32)	16 (26)	0.5462
History of diabetes mellitus (yes), n (%)	26 (22.4)	12 (21)	14 (23)	0.8276
Platelets (10^9^), median [IQR]	172 [136–211]	159 [135–204]	174 [140–220]	0.3186
ALT (IU/L), median [IQR]	22 [15.5–28]	22 [15.5–28]	21 [14.2–29]	0.2085
AST (IU/L), median [IQR]	23 [19–27]	21 [18–27]	23 [19–29]	0.1197
HCV-RNA before DAA (10^6^ IU/ml)	1.7 [0.8–3.3]	1.6 [0.9–3.5]	1.7 [0.5–3.1]	0.5932
HCV genotype, n (%)	—	—	—	0.3325
1	87 (75)	42 (75)	45 (75)	—
2	19 (16.4)	7 (12.5)	12 (20)	—
3	8 (6.9)	5 (8.9)	3 (5)	—
4	2 (1.7)	2 (3.6)	0	—

BMI: body mass index; ELF: enhanced liver fibrosis; TE: transient elastography; AHT: arterial hypertension; ALT: alanine aminotransferase; AST: aspartate aminotransferase; HCV: hepatitis C virus.

a Of the whole cohort, in 49% of the cases, the ELF score was not calculable.

Overall, the median baseline LSM was 9.6 [IQR 7.2–14] kPa. In particular, the LSM was 10.2 [IQR 7.2–15] kPa in the R group and 9.1 [IQR 7.3–12] kPa in the C group (*p* = 0.46). Overall, 29.3% of patients had history of arterial hypertension, and 22.4% of them had diabetes mellitus. The most frequent HCV genotype was 1 (75%) followed by genotype 2 (16.4%), without difference between groups. The median follow-up was 36.2 (IQR 31.4–41.7) months in the R group and 37.1 (IQR 25.7–46.6) in the C group. All but one patient who withdrew informed consent completed the treatment of 12 months in the R group.

At baseline (SVR12), 102 patients had Child–Pugh (CP) score A and 3 patients had CP score B. The variations of CP score before and after treatment are detailed in Supplementary Table S1.

### Transient Elastography and Enhanced Liver Fibrosis Variations

**FIGURE 2:**
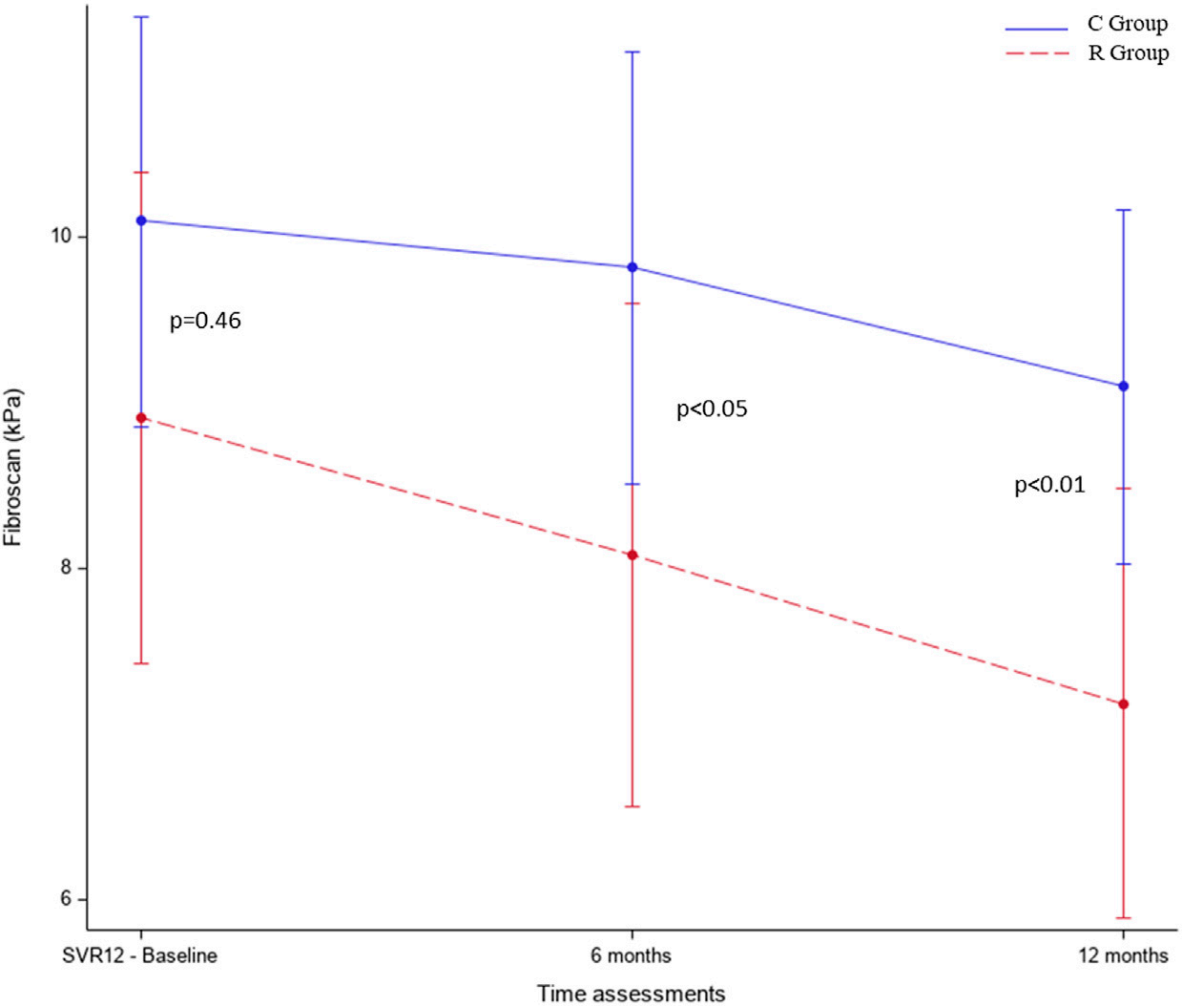
Liver stiffness variations in both groups between baseline and end of the study in the multivariate model adjusted for age, gender, cirrhosis, ALT, and AST. R group: patients who received active treatment; C group: patients with no intervention.

In both groups, LSM improved at 6 and 12 months in comparison with baseline. The multivariate model, adjusted for age, gender, cirrhosis, and AST and ALT parameters, showed that patients in the R group compared to those in the C group had a more significant improvement of LSM both after 6 (−2.05, 95% CI −3.89 to −0.22, *p* < 0.05) and 12 months of treatment (−2.79, 95% CI −4.5 to −1.09, *p* < 0.01) in comparison with baseline (Figure 2 and Table 2). Contrarily, no significant changes were observed in the ELF score after 12 months in the R group compared to the C group (ELF24-ELF12 diff = 0.4 kPa, *p* = 0.227) (Table 3).

**TABLE 2:** Results of multivariate MMRMs adjusted by age, gender, cirrhosis, and ALT and AST parameters. Estimated mean differences, and their 95% CI, between treatments and visits by MMRMs for transient elastography. The multivariate model includes treatment, visit, treatment and visit interaction, age, gender, the presence of cirrhosis, ALT, and AST. The result of MMRM analysis corresponds to the difference of the changes in transient elastography between the two arms at different time points.

Time point 1	Time point 2	Treatment	Treatment (ref.)	Difference in transient elastography (95%CI)	*p*-Value^a^
Baseline	Baseline	R group	C group	−1.19 (−3.03–−0.66)	0.2143
6m	6m	R group	C group	−1.74 (−3.65–−0.18)	0.2578
6m	Baseline	R group	C group	−2.02 (−3.89–−−0.15)	0.0258
12m	12m	R group	C group	−1.91 (−3.48–−−0.34)	0.0203
12m	6m	R group	C group	−2.63 (−4.38–−−0.89)	0.0122
12m	Baseline	R group	C group	−2.91 (−4.61–−−1.21)	0.0003

a Non-parametric test; 6m: 6 months (24 weeks); 12m: 12 months (48 weeks).

MMRM: multivariable linear mixed model of repeated measurements.

**TABLE 3:** ELF variations at baseline and 12 months after treatment.

	SVR12		12 months	
	R group	C group	R group	C group
ELF, median [IQR]	10.7 [10–11.2]	10.7 [9.5–11.2]	9.9 [9.2–11.1]	10.1 [9.3–10.8]

### Safety and Long-Term Outcome

No patient presented treatment-related adverse events. During the extended follow-up, no patients developed HCC in the R group, while the occurrence of HCC was observed in four patients of the C group (all patients with LSM >14 kPa, corresponding to F4 according to the METAVIR score). No case of liver decompensation neither variceal bleeding was reported in both groups. Four deaths were reported (2 in the R group and 2 in the C group) and were due to non-liver–related deaths in two patients.

## Discussion

The advent of DAA-based regimens for HCV infection, characterized by excellent efficacy and safety profiles, made possible HCV eradication also in patients with ACLD. Many studies showed that SVR achievement is clearly associated with the improvement of natural history and the reduction of liver-related events and mortality (D’Ambrosio et al., 2021; Di Marco et al., 2016; Van der Meer et al., 2017; Nahon et al., 2017). However, the issues regarding regression of liver fibrosis and portal hypertension remain controversial, especially in patients with cirrhosis and clinically significant portal hypertension (CSPH) (Rosso et al., 2020). Thus, the synergic action of additional molecules with anti-fibrotic effect, such as silybin, could have a relevant role.

In this multicenter, prospective, and interventional study, patients orally treated with a highly bioavailable form of silybin showed a more significant improvement of liver stiffness at 6 and 12 months compared to patients receiving no intervention after achieving SVR with DAAs. These preliminary results underline the plausible anti-fibrotic effect of silybin with consequent reduction of liver damage through the softening of inflammatory cascade and immune system modulation (Salomone et al., 2016). Moreover, silymarin seems to effectively interfere with fibrogenesis at different levels of the process. In a mouse model of CCl4-induced liver fibrosis, Clichici et al. (2015) demonstrated that the administration of silymarin for 4 weeks, at doses usually used in clinical practice as adjuvant in hypertransaminasemia, favors the reduction of hepatocyte damage, oxidative stress markers, and fibrosis score and the activation of both hepatic stellate cells (HSCs) and Kupffer cells.

Another anti-fibrotic effect of silybin is the antagonization of platelet-activating factor (PAF) action. Indeed, the PAF stimulates HSCs to produce a large quantity of collagen (Lu et al., 2008). This capacity of PAF would be opposed by the process of acetylation supported by lysophosphatidylcholine acyltransferase (LPCAT) enzymes, whose expression is clearly lower in cirrhotic patients compared to controls. In this regard, silybin is able to antagonize the profibrotic effect of PAF through the increase of LPCAT expression, as well as through a direct reduction of PAF in cirrhotic Wistar rats (Stanca et al., 2013). Moreover, in a study on rats, treatment with silybin reduced the production of collagen-I and *α*-SMA (alpha-smooth muscle actin), with consequent inhibition of HSC activation (Hu et al., 2019). Of note, it has been demonstrated that silybin is able to reduce liver fibrosis in NAFLD patients with paired liver biopsy before and after treatment (Loguercio et al., 2012).

In our cohort, with a median follow-up of 23 months, four patients developed HCC, all in the control group. This finding could be further analyzed in a larger cohort of patients considering the potential anti-cancer modulatory effect of silybin. Indeed, silybin could interfere with the tumoral process through inflammatory cascade regulation and by decreasing the ROS genotoxic potential. Moreover, it could block most of the signaling pathways activated in HCC (Gopalakrishnan et al., 2013; Mao et al., 2018).

The present study has some limitations. First, the assessment of LSM with transient elastography after DAA therapy carries a lower reproducibility in comparison with its pre-DAA value (European Association for the Study of the Liver 2021). Moreover, this is a pilot study with a small number of patients, and especially, the long-term outcomes should be further assessed in larger studies to confirm these results.

In conclusion, in our study, we showed that the combination of silybinphospholipid complex with vitamin D and vitamin E (Realsil 100D^®^, Ibi Lorenzini S.p.A., Aprilia, Italy) is potentially able to favor the regression of liver fibrosis in patients with SVR after DAA treatment for HCV infection. Larger studies are needed to carefully evaluate how this could potentially impact on liver-related outcomes.

## Data Availability

The raw data supporting the conclusions of this article will be made available by the authors, without undue reservation.
