# Supplementary material for: Beneficial Effects of Silybin Treatment After Viral Eradication in Patients With HCV-Related Advanced Chronic Liver Disease: A Pilot Study
Source: Front Pharmacol. 2022 Feb 2;13:824879. doi: 10.3389/fphar.2022.824879 (PMC8847679; doi:10.3389/fphar.2022.824879)
Supplement: Supplementary file 1 [file Table1.DOCX]

Supplementary Tables

**Table 1.** Variations of Child-Pugh score at baseline, 6 months and 12 months after treatment

|  | **Baseline** | | **6 months** | | **12 months** | |
| --- | --- | --- | --- | --- | --- | --- |
| Child-Pugh Score, n (%)  A  B | R Group | C Group | R Group | C Group | R Group | C Group |
|  | 44 (43.1)  1 (33.3) | 58 (56.9)  2 (66.7) | 44 (44)  1 (50) | 56 (56)  1 (50) | 43 (43.9)  1 (33.3) | 55 (56.1)  2 (66.7) |
| Non-cirrhotic | 11 | 0 | 11 | 3 | 12 | 3 |
